# Removal of a Fish Bone Completely Embedded in the Cervical Esophageal Wall

**DOI:** 10.7759/cureus.93514

**Published:** 2025-09-29

**Authors:** Kenichi Watanabe, Yuho Sato, Yuichi Shimizu, Yutaro Saito, Kiyoshi Oda

**Affiliations:** 1 Otolaryngology, Tohoku Rosai Hospital, Sendai, JPN

**Keywords:** cervical incision, endoscopic ultrasound, esophageal foreign bodies, fish bone, intraoperative, neck exploration, ultrasonography, ultrasound

## Abstract

Fish bone is one of the most frequent foreign bodies in the upper aero-digestive tract. When esophageal foreign bodies penetrate outside the lumen or migrate to the extraluminal region, removal through a neck exploration should be considered. We report a rare case of a 68-year-old woman in whom an ingested fish bone became completely lodged in the cervical esophageal wall. First, an esophagogastroduodenoscopy was performed, followed by a rigid esophagoscopy under general anesthesia; however, no foreign bodies or abnormalities in the esophageal mucosa were found. Endoscopic ultrasound was performed with the assistance of a bivalve pharyngoscope to dilate the entrance to the esophagus, but it was not possible to accurately identify the fish bone that had completely embedded in the esophageal muscle layer. Finally, a cervical incision was made, and the location of the fish bone foreign body was confirmed by directly applying an ultrasound linear probe to the exposed esophageal wall. An incision was then made in the esophageal wall, and the fish bone was removed. Because it is pretty challenging to remove foreign bodies that have completely lodged in the esophageal wall, multiple removal methods should be prepared. We recommend using an intraoperative ultrasound device to identify the location of foreign bodies in the neck.

## Introduction

Fish bone ingestion is one of the most frequent foreign bodies in the upper aero-digestive tract, particularly in East Asia, because a whole fish is often served without removing its bones. Although fish bone foreign bodies are not frequently associated with severe complications, they can cause several life-threatening complications, including perforation of the esophagus, deep neck infection/abscess, mediastinitis/mediastinal abscess, and aortoesophageal fistula [1]. Therefore, prompt diagnosis followed by adequate treatment is required to manage fish bone foreign bodies in the upper aero-digestive tract [1]. Recently, most cases of esophageal foreign bodies can be removed endoscopically [2]; however, if they penetrate outside the lumen or migrate to the extraluminal region, removal through a neck exploration should be considered. We report an extremely rare case of a fish bone completely embedded in the cervical esophageal wall that required multiple removal methods.

## Case presentation

**Figure 1:**
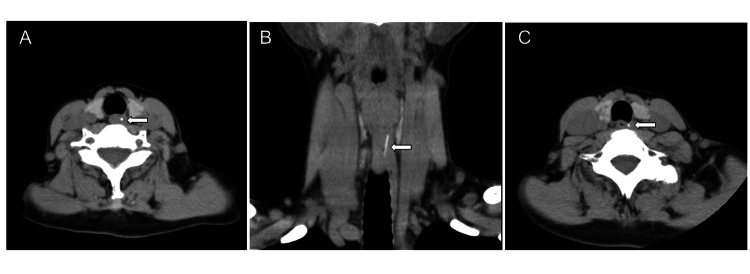
(A) Axial CT of the neck showing the fish bone (arrow) in the esophagus. (B) Coronal CT of the neck showing the fish bone (arrow) positioned obliquely downward. (C) A neck CT scan taken 19 days later showing the fish bone in nearly the same position as the previous images.

A 68-year-old woman who experienced a pricking sensation for three days after eating boiled red fish was admitted to a local hospital. A general examination could not find a fish bone in her oral cavity and laryngo-pharynx, but computed tomography (CT) showed a high-density linear shadow in the cervical esophagus (Figure 1A, 1B).

**Figure 2:**
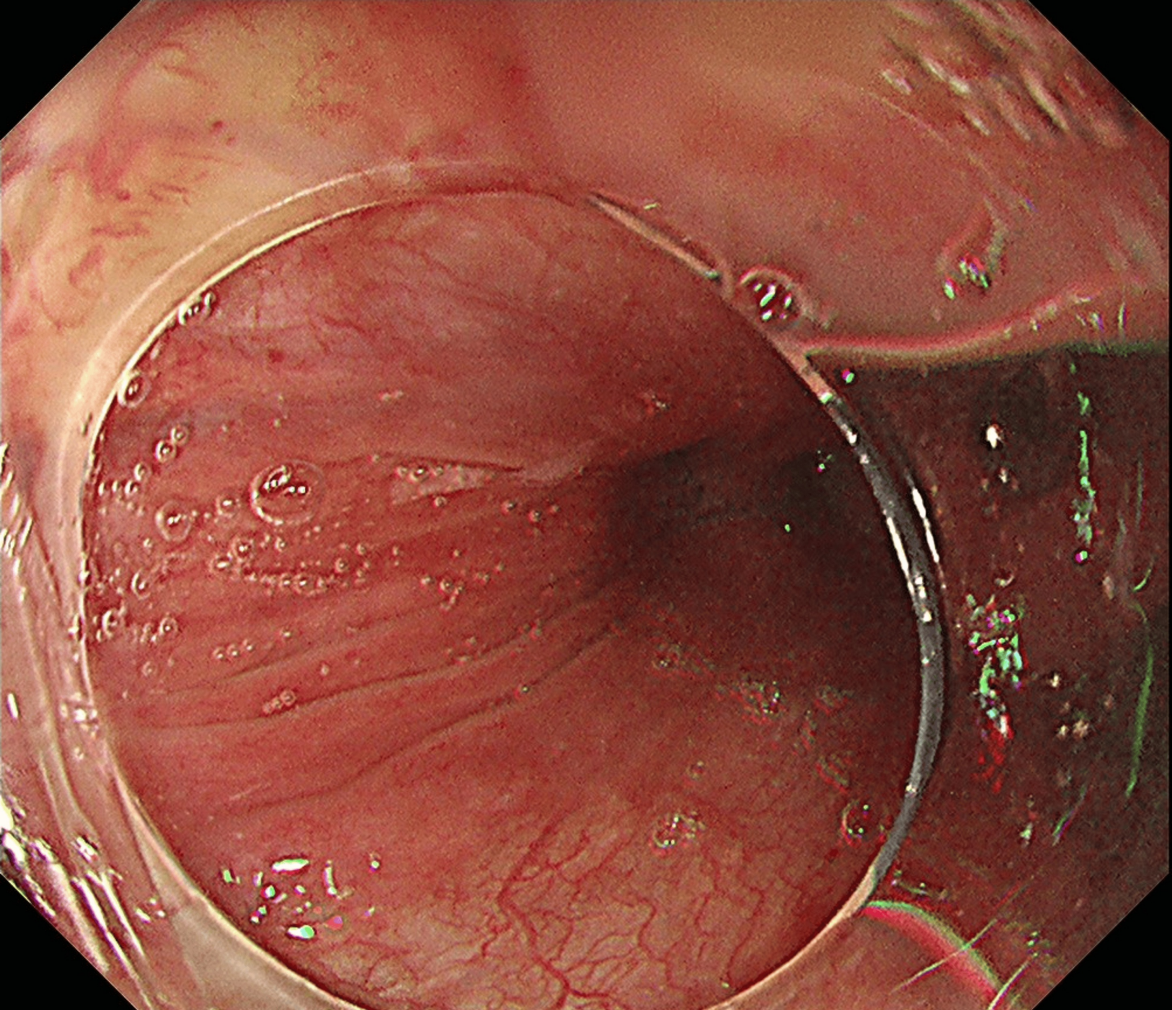
Esophagogastroduodenoscopy showing smooth surface without redness or swelling in the cervical esophageal mucosa.

The patient was referred to our hospital for the removal of the fish bone. Firstly, gastrointestinal endoscopists at our hospital performed an esophagogastroduodenoscopy (EGD) and found no foreign body, including a punctured fish bone. No redness or swelling of the esophageal mucosa was observed (Figure 2).

The next day, we otolaryngologists performed rigid esophagoscopy under general anesthesia; however, we were unable to identify the fish bone or even the abnormal mucosa. The patient’s symptoms of pain on swallowing had resolved postoperatively. CT taken 19 days later showed the linear high-density material as in the previous image (Figure 1C); thus, we considered the foreign body to be completely embedded in the esophageal wall.

**Figure 3:**
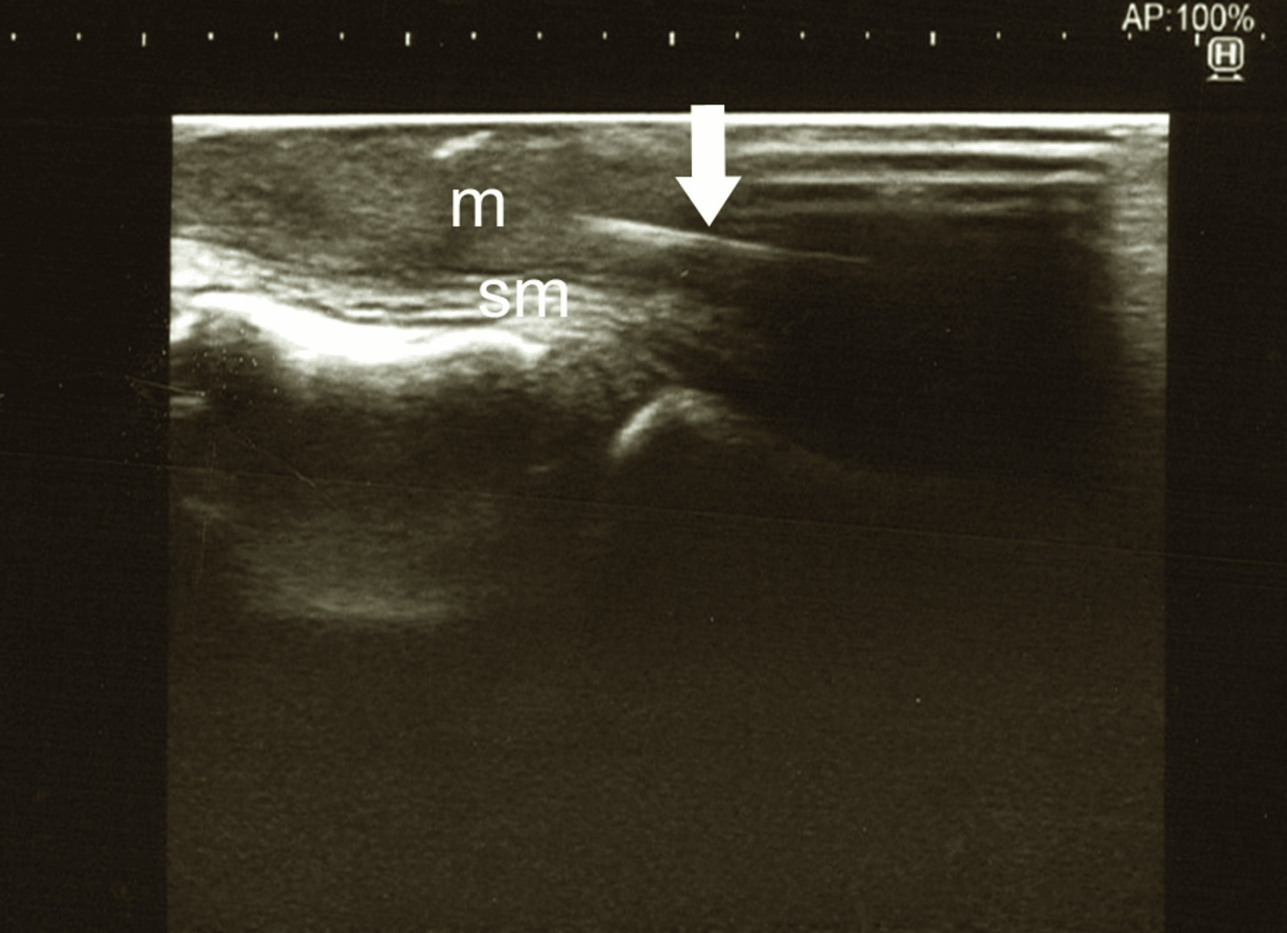
An ultrasound examination using a 13–6 MHz linear probe (Sonosite SⅡ, FUJIFILM, Tokyo, Japan) revealed the fish bone (arrow) located within the muscular layer of the esophageal wall. sm, submucosal layer; m, muscular layer

**Figure 4:**
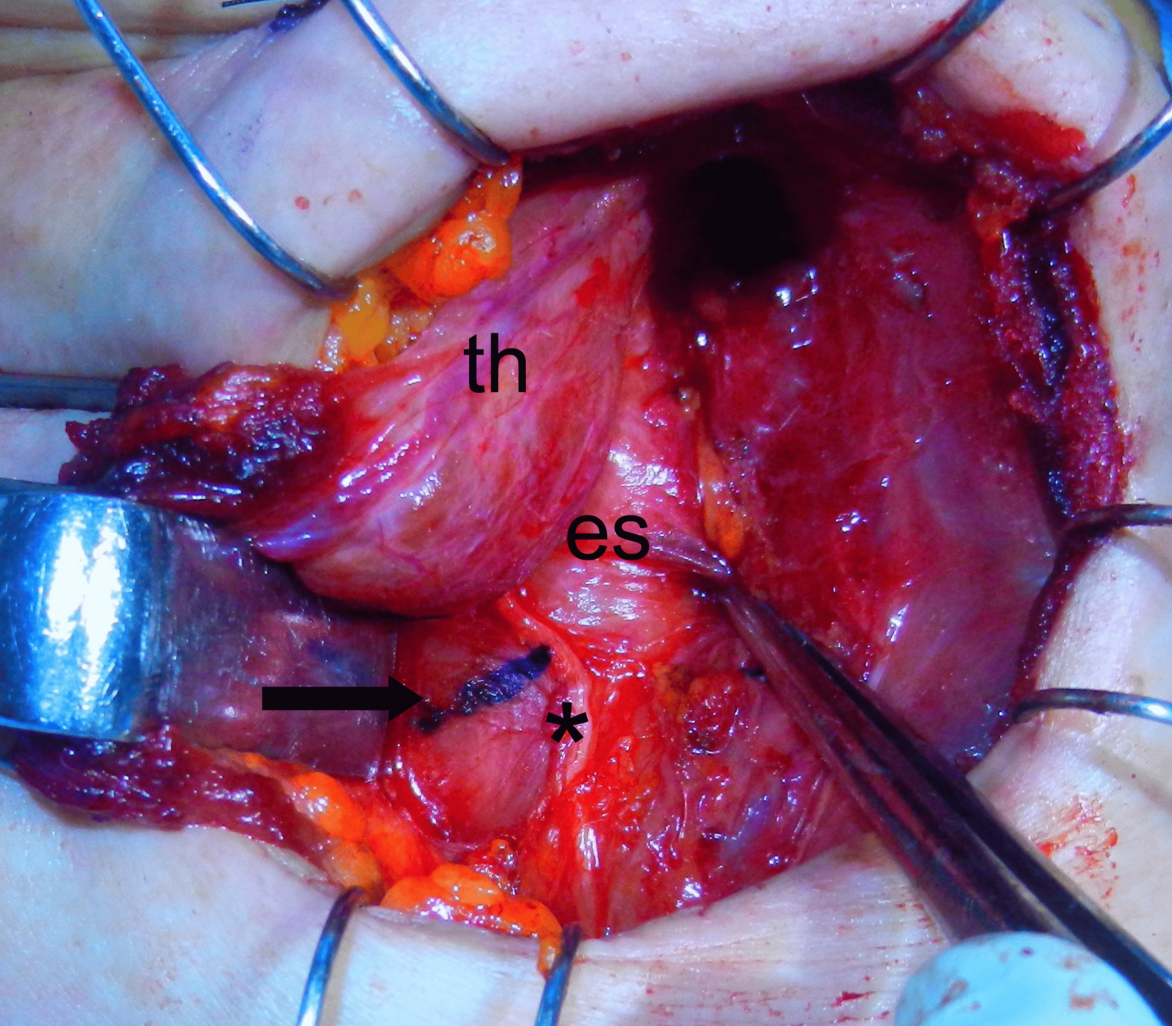
Intraoperative view of the esophageal surface in the neck. The incision site marked in blue (arrow) on the esophageal wall was confirmed by intraoperative ultrasonography. *, left recurrent neve; th, left lobe of the thyroid gland; es, esophagus

**Figure 5:**
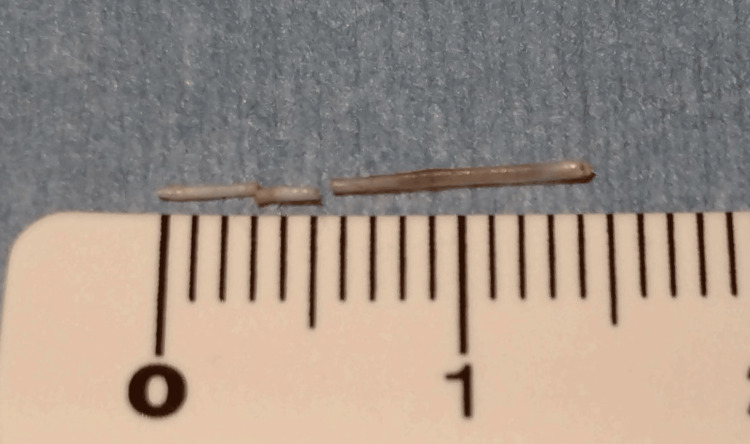
Fish bone removed in 3 sections, which was 14 mm in length.

Twenty-eight days after the initial visit, we, otolaryngologists and endoscopists, cooperated to perform the surgery under general anesthesia; a bivalve Weerda pharyngoscope was inserted orally at the hypopharynx and cervical esophagus to support widening the lumen, then, endoscopic ultrasound (EUS) was carried out to explore the fish bone embedded in the esophageal wall. In practice, EUS failed to delineate the fish bone clearly in the esophageal wall. With the assistance of ultrasound (US) examination from the neck surface, two mucosal incisions were made in the short-axis direction with a dual knife, targeting an indistinct high-echoic structure by EUS, but no submucosal foreign body could be identified. We considered that most of the fish bone had migrated into the muscular layer, abandoned the endoscopic approach, and decided to perform the removal method through an external cervical incision. The left sternothyroid muscle was cut, the middle thyroid vein and the inferior thyroid artery were ligated. The left lobe of the thyroid gland was translated ventrally, and the left recurrent nerve was confirmed using the NIM-Neuro™ 3.0 nerve monitoring system (Medtronic, Minneapolis, MN, USA). After the esophageal wall and recurrent nerve were exposed, a US linear probe was applied directly to the esophageal wall to identify the fish bone located within the muscular layer of the esophageal wall, which could not be confirmed by palpation (Figure 3). The recurrent nerve was slightly compressed laterally, and a 1 cm short-axis incision was made through the esophageal muscular layer (Figure 4). Careful exploration with mosquito forceps identified a 9 mm long fish bone foreign body, and it was removed gently. The remaining 2 mm and 3 mm lengths of the broken fish bones were subsequently removed, and the total length was about 14 mm, so we considered the entire fish bone removed (Figure 5).

There was no abscess formation within the incised muscle layer. After the wound was washed with saline solution, the esophageal muscular layer was sutured with 3-0 PDS™. No serious complications occurred postoperatively. Left vocal cord paralysis developed, but the patient fully recovered three months later.

## Discussion

Fish bone foreign bodies in the upper aero-digestive tract are a common presentation in the emergency department and otolaryngology clinics, especially in countries with high rates of fish consumption, such as Asian, Mediterranean, and other coastal countries [1,3]. If accidentally swallowed, fish bones are commonly found impacted in the palatine tonsils, lingual tonsils, or vallecula, where they can be easily removed in an outpatient procedure. Other sites, such as the pyriform sinus or posterior wall of the hypopharynx, and the cervical esophagus, can also be involved [1,3]. When encountering a case of a fish bone foreign body in the esophagus, the existing removal methods include endoscopic removal by EGD under local anesthesia, using a rigid esophagoscope under general anesthesia, or surgical intervention such as cervical external incision. If the fish bone can be removed endoscopically, it is less invasive and preferable for the patient than the neck incision method. However, when the ingested foreign body became completely embedded in the esophageal wall, it would be difficult to find and remove. Koito reported a case of an 83-year-old woman in whom a fish bone foreign body was completely embedded in the cervical esophageal wall, but the upper gastrointestinal endoscope confirmed the mucosa with erosion, redness, and edema, and removed it through an endoscopic incision [4]. The author described that endoscopic removal should be the first choice; however, it does not apply to cases in which it is clear that a completely embedded foreign body has damaged the muscle layer [4].

Recently, there have been several reports of the use of EUS to precisely identify the location of fish bones that have entirely migrated into the esophageal wall during their removal operations [5-8]. Cao et al. described that the usefulness of EUS in diagnosing foreign bodies is promising, especially for those located in unconventional positions such as the esophageal submucosa [6]. Wang et al. reported the importance of accurately locating the fish bone position by EUS [8]. In our case, an attempt to identify the fish bone using EUS yielded only uncertain images, resulting in an unsuccessful endoscopic incisional removal, and the decision was made to transition to an external incision. It is unclear why the EUS could not delineate the target object, but one reason may be that the embedded fish bone was thin, which was less than 1 mm wide. Another reason may be that the fish bone had migrated deep into the muscular layer rather than the submucosa.

Conventional US imaging from the neck surface is still useful both for preoperative evaluation and as real-time images during surgery. There have been reports that cervical US assistance was helpful when endoscopically removing fine bone foreign bodies in the mucosa of the upper esophagus [9,10]. Even when performing surgical neck exploration, US has been reported to be available transcutaneously as an intraoperative guide by providing real-time images [11,12], and is also reported to be useful when applied directly within the wound [11,13]. In our case, the fish bone foreign body could not be identified visually or palpated, even in the esophageal wall exposed by an external cervical incision. US imaging served as an indispensable guide for the incision of the esophageal wall. In a paper describing the usefulness of using computer-based imaging for removing foreign bodies in the head and neck region, the author concluded that image-guided surgery helps to prevent major complications, including injury to surrounding vital structures, shortens the operating time, and allows minimally invasive access [14]; that is true for using intraoperative US as well. We recommend the use of US for locating migrated foreign bodies in the neck.

## Conclusions

Removal of a foreign body that is completely embedded within the esophageal wall, as in our case, can be pretty challenging. Multiple removal methods should be prepared and handled in the extraction process, with consideration for patient safety and meticulous surgical technique. The use of intraoperative US during an external cervical incision serves as *a light to guide* the surgeon *in the darkness*.
